# Interaction between endothelial injury and immune response in septic shock: from basic research to clinical applications

**DOI:** 10.3389/fphys.2025.1627008

**Published:** 2025-09-04

**Authors:** Yang Wang, Qing-Nan Guan, Zhong-Jv Zhang, Yu-Meng Zhang

**Affiliations:** ^1^ Department of Pleurisy, The Tenth People’s Hospital of Shenyang, Shenyang, China; ^2^ Intensive Care Unit, The Tenth People’s Hospital of Shenyang, Shenyang Chest Hospital, Shenyang, China

**Keywords:** septic shock, immune dysregulation, endothelial injury, clinical applications, endothelium-targeted therapy

## Abstract

Septic shock is a life-threatening condition caused by microorganisms and their toxins, which often results in severe haemodynamic instability and multi-organ dysfunction. Immune system dysfunction and endothelial injury play crucial roles in its pathogenesis and progression. In septic shock, pathogen recognition triggers immune activation, leading to excessive cytokine release and hyperactivation of immune cells. This overwhelming inflammatory response not only exacerbates endothelial injury, but also increases the risk of secondary infections, creating a vicious cycle that suppresses immune function and increases mortality. Cytokines alter the endothelial cell phenotype and structure, causing dysfunction, increased vascular permeability, and infiltration of inflammatory cells and cytokines into the interstitial space. The exposure of adhesion molecules promotes leukocyte migration and activation of coagulation pathways, significantly increasing the risk of thrombosis. These interactions contribute towards systemic oedema, hypotension, and microcirculatory dysfunction, exacerbating organ hypoxia and failure. This article explores the intricate interplay between endothelial injury and immune response in septic shock and its clinical implications. We highlight the potential of immunomodulation in mitigating immune damage as well as suppression. Additionally, we discuss endothelium-targeted therapies, including anti-inflammatory strategies, endothelial repair, and microcirculation improvement. Future research should focus on developing novel drugs and refining therapeutic approaches to effectively counteract endothelial damage and immune dysregulation, ultimately improving clinical outcomes and reducing morbidity and mortality.

## 1 Introduction

Sepsis is a life-threatening organ dysfunction caused by a dysregulated host response to infection. It affects >30 million people worldwide each year, has a mortality rate of up to 10%, and is a leading causes of death among critically ill patients worldwide (Girardis et al., 2024). Septic shock represents an extreme and fatal stage of sepsis characterised by severe circulatory failure and abnormal cellular and metabolic functions (Singer et al., 2016). It manifests as haemodynamic instability and is unresponsive to fluid resuscitation. Clinically presenting with systemic hypotension, microcirculatory dysfunction, multi-organ failure, tissue hypoxia, and cellular metabolic disturbances, it constitutes a complex critical condition. With a mortality rate as high as 40%–60%, septic shock is the leading cause of death among patients with sepsis, with a patients (Font et al., 2020; Wu et al., 2020).

Abnormal immune activation and endothelial injury are hallmark pathological changes in septic shock that mutually promote the worsening of the disease process (McDonald et al., 2017). Excessive activation of immune responses and sustained inflammatory reactions are major drivers of the onset and progression of septic shock. Neutrophils primarily counteract pathogens by phagocytosis, degranulation, and the release of neutrophil extracellular traps (NETs) (Kienle et al., 2021). However, the overactivation of immune cells and abnormal secretion of cytokines induce endothelial cells to adopt a pro-inflammatory and procoagulant phenotype, thereby increasing vascular permeability (Alsabani et al., 2022). Consequently, the endothelial barrier collapses, leading to microcirculatory disturbances, peripheral hypovolaemia, and organ failure during late septic shock (Joffre et al., 2020).

Numerous studies have demonstrated that the complex interplay between vascular endothelial injury and the immune response makes sense not only in the pathogenesis and progression of septic shock but also offers new insights for clinical treatment (Zhang et al., 2023). Modulating the immune response and restoring endothelial function may represent emerging therapeutic strategies for septic shock.

This review discusses the role of immune responses and endothelial injury in septic shock and their underlying mechanisms, combining basic research with clinical applications to provide new perspectives for the early diagnosis and treatment of septic shock and promote the clinical translation of related intervention strategies.

## 2 Pathophysiology of septic shock

**FIGURE 1:**
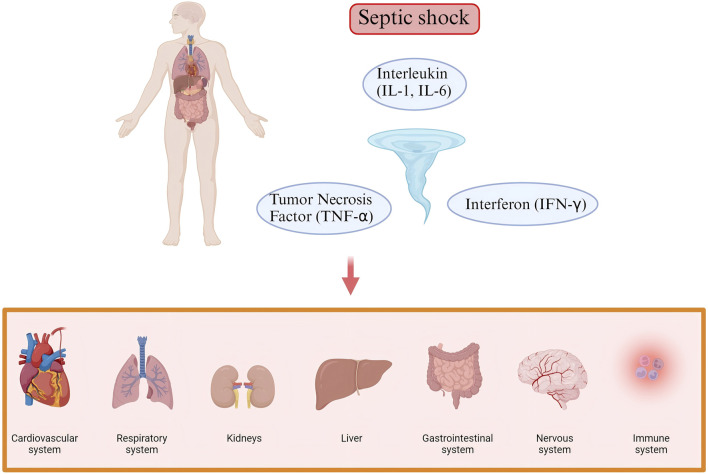
The complex pathogenesis of Septic shock. The pathophysiological mechanisms of septic shock involve widespread dysregulation of multiple systems, including the cardiovascular system, respiratory system, renal system, hepatic system, gastrointestinal system, nervous system, and immune system.

The pathophysiological mechanisms of septic shock are highly complex and involve involve widespread dysregulation of multiple systems, including the cardiovascular system, respiratory system, renal system, hepatic system, gastrointestinal system, nervous system, and immune system (Figure 1). The core issue lies in the systemic inflammatory response triggered by infection, accompanied by immune dysregulation, vascular dysfunction, and microcirculatory disturbances among a series of physiological changes (Girardis et al., 2024). A delicate balance between pro- and anti-inflammatory responses is crucial in this process.

When bacteria, viruses, fungi, and other pathogens invade the body, they express conserved sequences known as pathogen-associated molecular patterns (PAMPs) such as bacterial lipopolysaccharides (LPS), flagellin, and lipoteichoic acid, as well as viral RNA and DNA (Kawai and Akira, 2010).

During sepsis, inflammatory responses or dead cells generate damage-associated molecular patterns (DAMPs) and release these molecules into the extracellular environment. This release further activates innate immune cells and triggers cytokine production (Lamkanfi, 2011). Pattern recognition receptors (PRRs) recognise and bind to PAMPs and DAMPs, initiating immune responses (Gregorius and Brenner, 2023). During this process, macrophages release large amounts of cytokines, including tumour necrosis factor (TNF-α), interleukins (IL-1, IL-6), and interferons (IFN-γ) (Guo et al., 2018). Common PRRs include Toll-like receptors (TLRs) and nucleotide-binding oligomerisation domain-like receptors. PRRs on immune and endothelial cells activate downstream signalling pathways such as nuclear factor kappa B (NF-kB) and activator protein 1, promoting the release of cytokines (Hayden and Ghosh, 2011). These cytokines mediate the immune-inflammatory response to either damage or inhibit pathogen growth. At the same time, it activates endothelial cells, triggering the coagulation pathway, forming microvascular thrombi, and preventing the systemic spread of pathogens (Pons et al., 2020). To provide proper checks and balance, cells produce anti-inflammatory cytokines (e.g., IL-10) and other mediators (e.g., prostaglandins) to properly control the inflammatory response (Ginhoux et al., 2016). Research has uncovered that extracellular histones directly activate NLRP3 inflammasomes within endothelial cells, driving pyroptotic cell death and consequently triggering endothelial dysfunction and immune dysregulation. Circulating histone levels strongly correlate with pyroptosis-associated factor expression, endothelial adhesion molecule release, and the severity of septic shock (Beltran-Garcia et al., 2022a). Beyond their inflammatory role, extracellular histones profoundly impact coagulation. Studies demonstrate histones can obliterate activated protein C’s protective effect on endothelium, shielding cells from thrombin-induced hyperpermeability and thereby fostering a prothrombotic milieu (Mayer et al., 2015). Furthermore, damage-associated molecular patterns (DAMPs) ignite systemic inflammation, platelet activation, coagulation cascade initiation, and fibrinolysis impairment—all processes intimately linked to endothelial injury and culminating in anticoagulant system failure. This represents a core pathophysiological mechanism driving disseminated intravascular coagulation (DIC) (Gando et al., 2024). Critically, extracellular histones are closely associated with the prognosis of septicemia patients.

Under normal circumstances, the immune response is self-regulating; however, in septic shock, the immune response is severely dysregulated. The inflammatory response is excessively activated, causing immune cell dysfunction and an inability to effectively clear pathogens; however, the immune system may enter an immunosuppressive state, leading to an increase in immunosuppressive cells, further aggravating the spread of infection and creating a vicious cycle (Beltran-Garcia et al., 2022b). Excessive cytokine reactions can trigger a ‘cytokine storm’, characterised by a rapid increase in cytokine concentrations. Under the activation of inflammation, endothelial cell dysfunction impairs the normal constriction and dilation of blood vessels, leading to endothelial cell injury. This not only affects vascular permeability but also promotes coagulation and exacerbates thrombus formation. Thrombus formation and embolism in microvessels can lead to blood flow obstruction, causing local tissue hypoxia and worsening microcirculatory dysfunction. Moreover, microcirculatory disturbances hinder the effective delivery of oxygen and nutrients to tissue cells, exacerbating local hypoxia, acidosis, and other pathological changes. Additionally, this leads to a significant drop in blood pressure, hypovolaemia, inadequate organ perfusion, and, ultimately, multi-organ failure (Girardis et al., 2024).

Therefore, the treatment of septic shock requires not only control of the infection source but also precise regulation of immune responses, vascular function, and microcirculatory disturbances to restore organ function and reduce mortality.

## 3 Immune response in septic shock

**FIGURE 2:**
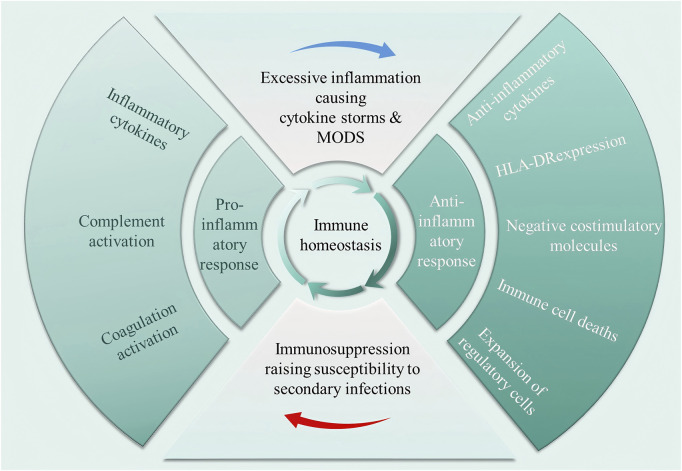
Dysregulation of immune homeostasis occurring in septic shock. In a normal physiological state, pro-inflammatory and anti-inflammatory responses maintain a dynamic balance. Septic shock disrupts this balance, triggering the massive release of pro-inflammatory cytokines, activating the complement and coagulation systems, leading to excessive inflammation, cytokine storms, and multiple organ dysfunction syndrome (MODS). At the same time, the enhanced anti-inflammatory response induces immune suppression, significantly increasing the risk of secondary infections.

Pathogens commonly isolated from patients with septicaemia include bacteria, fungi, parasites, or viruses, with bacteria being the most common (Martin et al., 2003). Upon pathogen invasion, the host’s innate immune system is activated as PRRs recognise PAMPs and DAMPs (Wiersinga et al., 2014). Both pro- and anti-inflammatory cytokine storms occur early during infection, and the balance between them determines whether excessive inflammation or immune suppression occurs clinically (Xiao et al., 2011). If the immune system effectively clears pathogens during the early stages of invasion, immune homeostasis can be rapidly restored. Conversely, patients with immune dysregulation are susceptible to secondary infections, prolonged immunosuppression, and ultimately the development of septic shock. As illustrated in Figure 2, immune homeostasis plays an important role in the pathophysiology and clinical outcome of septic shock. Under normal physiological conditions, a dynamic balance between pro-inflammatory and anti-inflammatory responses maintains immune homeostasis. This balance is disrupted when septic shock occurs. Inflammatory cells release pro-inflammatory cytokines in large quantities, which in turn activate, for example, the complement system and the coagulation system, leading to excessive inflammation, which further triggers a cytokine storm and multiple organ dysfunction syndrome (MODS). Increased release of anti-inflammatory cytokines and synergistic inhibitory molecules, decreased human leukocyte antigen DR isotype (HLA-DR) expression, immune cell death, and expansion of regulatory cells leading to immunosuppression increase the susceptibility to secondary infections, which is the main reason for the poor prognosis of patients with sepsis.

### 3.1 Activation of immune cells and inflammatory response

In septic shock, the inflammatory response is primarily mediated by inflammatory factors and neutrophils. Cytokines known to play significant roles in modulating the host response in septic shock include TNF-α, IL-1, IL-6, IFN-γ, granulocyte colony-stimulating factor, macrophage migration inhibitory factor (MIF), and HMGB-1. TNF-α and IL-1 are the most extensively studied pro-inflammatory cytokines, while MIF regulates immune responses by modulating TLR4 (Roger et al., 2001), and the pro-inflammatory cytokine HMGB-1 has been identified as a late mediator of septic shock (Sunden-Cullberg et al., 2005). NETs are DNA-decondensed fibres that carry citrullinated histones and proteolytic enzymes, such as myeloperoxidase and neutrophil elastase. Plasma from patients with septic shock reportedly induces platelet–neutrophil interactions in a TLR4-dependent manner, resulting in the formation of NETs (Clark et al.). Apart from inflammatory factors and NETs, the complement system (Author Anonymous, 1989), coagulation system (Machado and Silva, 2006), endothelial cells (Chang, 2019), and autonomic nervous system interact with each other and play important roles in immune system activation and inflammatory responses.

### 3.2 Overreaction of immune cells and immune suppression

The immune response in septic shock is a dynamic and time-dependent process. Overactivation of the immune system leads to the compensatory anti-inflammatory response syndrome (CARS), and when CARS and systemic inflammatory response syndrome coexist, patients with septic shock enter a state of immunosuppression. The main manifestations are the massive release of anti-inflammatory cytokines, depletion and death of T cells and other immune cells, and massive activation of regulatory T cells (Tregs) and myeloid-derived suppressor cells (MDSCs) that play an immunomodulatory role.

The major anti-inflammatory cytokines associated with septic shock include IL-4, IL-10, and IL-37, which are secreted by various immune cells. These anti-inflammatory cytokines suppress the release of pro-inflammatory cytokines, antigen presentation, and T-cell proliferation and function, while promoting the proliferation and differentiation of immunosuppressive cells such as Tregs and MDSCs (Hotchkiss et al., 2013). Inflammatory factors can induce cell death in monocytes, macrophages, and T cells via various mechanisms. Common forms of cell death, such as apoptosis (Luan et al., 2015; Li et al., 2016), pyroptosis (Wang et al., 2018; Deng et al., 2018), autophagy (Park et al., 2017), and ferroptosis (Wen et al., 2019), can also contribute to immune suppression induced by septic shock. Additionally, metabolic paralysis of immune cells is another hallmark of immune suppression (Cheng et al., 2016). Apart from the dysfunction or loss of immune effector cells, excessive activation of immunoregulatory cells also contributes to immune suppression in septic shock. Tregs induce immune suppression by releasing anti-inflammatory cytokines such as transforming growth factor (TGF)-β and IL-10, and upregulating immune checkpoint molecules on immune effector cells, including T-cell immunoglobulin and mucin-domain containing-3 (TIM-3) (Huang et al., 2022), programmed cell death protein 1 (PD-1) (Chen and Zhou, 2021), and cytotoxic T-lymphocyte antigen-4 (Mewes et al., 2019). MDSCs contribute primarily to chronic and sustained immune suppression in patients with sepsis (Liu et al., 2022).

## 4 Role of endothelial injury in septic shock

Endothelial cells are unconventional immune cells that maintain the integrity of the vascular wall through tight junctions and intercellular adhesion molecules, regulate vascular permeability, and participate in systemic immune responses following infection. They help limit the spread of pathogens (Ince et al., 2016). In septic shock, the endothelial responses triggered by various PAMPs and DAMPs are polymorphic, heterogeneous, and multifaceted. Inflammatory stimuli lead to glycocalyx damage and increased vascular permeability, causing leakage of fluids and proteins into the tissue interstitium, resulting in oedema and hypovolaemia. This fluid loss leads to decreased blood pressure and inadequate organ perfusion, further exacerbating the clinical manifestations of shock. Simultaneously, low blood volume and slow blood flow can lead to organ hypoxia and dysfunction, thereby increasing mortality rates (Opal and van der Poll, 2015).

### 4.1 Glycocalyx injury

The glycocalyx is a gel-like layer on the luminal surface of endothelial cells and is composed of membrane-bound proteoglycans, glycoproteins, glycosaminoglycans, and plasma proteins that adhere to the vessel walls (Weinbaum et al., 2007). Under homeostasis, the glycocalyx performs vascular barrier functions, participates in haemostasis, facilitates leukocyte and platelet adhesion, and transmits shear stress to endothelial cells (Alphonsus and Rodseth, 2014). In septic shock, neutrophils, NETs, and their induced pro-inflammatory cytokines, such as TNF-α, IL-6, and IL-8 (Ma et al., 2019), damage the glycocalyx. Additionally, various inflammatory cells and factors contribute to glycocalyx degradation and shedding, such as cytokines, proteases, histamine, and heparinases released by mast cells (Ferrario et al., 2019), histone deacetylases induced by oxidative stress (reactive oxygen species [ROS]), and matrix metalloproteinases (MMPs) (Ali et al., 2019).

### 4.2 Endothelial cell damage and increased vascular permeability

Glycocalyx disruption exposes endothelial cells, and various pro-inflammatory factors such as TNF-α, heparinase, and NETs, through binding to receptors on the endothelial cell surface, activate downstream signalling pathways, inducing an increase in endothelial cell shedding products and dysfunction (Nieuwdorp et al., 2009). Additionally, the release of cytokines promotes endothelial cell apoptosis and inhibits endothelial cell regeneration, further exacerbating vascular injury. Shedding of the glycocalyx may also lead to an increased release of nitric oxide (NO) or endothelin (ET) by endothelial cells, direct membrane damage caused by lipid peroxidation induced by ROS, and the breakdown of tight junction molecules that anchor endothelial cells together, impairing vascular contractile function due to mechanical dysfunction (Bolon et al., 2008). These factors further increase vascular permeability, allowing plasma components to leak into the tissue interstitium, which in turn causes oedema and hypovolemia (Rubio-Gayosso et al., 2006). Furthermore, cell junction proteins, such as vascular endothelial cadherin (VE-cadherin), occludin, and zonula occludens-1 (ZO-1), play a central role in maintaining interendothelial cell connections and regulating vascular permeability. The expression and function of these proteins are regulated by various factors, including inflammatory mediators, metabolites, and signalling pathways. First, as a core component of the endothelial adherens junctions, the stability of VE-cadherin is crucial for maintaining vascular endothelial barrier integrity. In septic shock, the expression and function of VE-cadherin are often disrupted. Neutrophil elastase and metalloproteinases hydrolyse the extracellular domain, thereby increasing vascular endothelial permeability. Studies have shown that lactate promotes VE-cadherin proteolysis *via* the extracellular signal-regulated kinase-dependent calpain activation pathway, significantly increasing vascular permeability and exacerbating organ dysfunction (Kun Yang et al., 2022). Additionally, S100A8/A9 protein deficiency can alleviate sepsis-induced lung inflammation and increase vascular permeability, an effect associated with the restoration of VE-cadherin expression (Yu et al., 2023). Second, occludin and ZO-1 are critical for endothelial tight junctions. Deficiency or inhibition of PLD2 protects the expression of tight junction proteins such as claudin-5, occludin, and ZO-1 by regulating the phosphatidic acid/signal transducer and activator of transcription three phosphorylation pathway, thereby mitigating sepsis-associated acute lung injury (Qian et al., 2023). Sulodexide improves sepsis-related endothelial barrier dysfunction by inhibiting glycocalyx degradation and upregulates ZO-1 expression *via* the NF-κB/ZO-1 pathway (Ying et al., 2023). Third, signal transduction significantly influenced the regulation of vascular permeability. The ARF6 inhibitor NAV-2729 enhances vascular integrity by restoring VE-cadherin expression, while inhibiting the phagocytosis of ASC specks to reduce inflammatory spread, thereby protecting against septic lung injury (Xiao et al., 2024).

These findings strongly indicate that the interaction between cell junction proteins and signalling pathways is of core significance for maintaining vascular endothelial connections and regulating permeability in septic shock.

## 5 Interaction between immune response and endothelial injury

**FIGURE 3:**
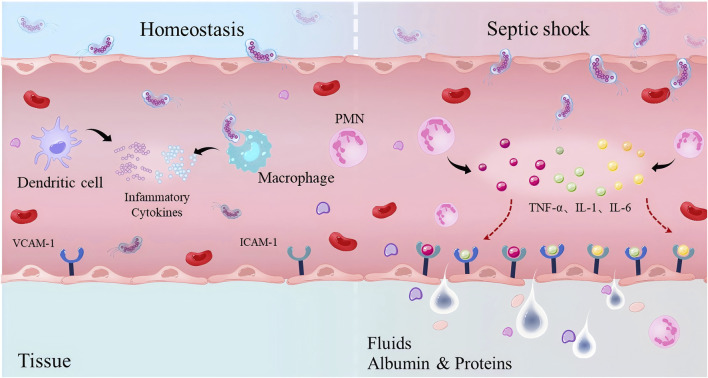
The endothelial cell injury induced by inflammatory factors. Left: Under homeostasis, it maintains local selective permeability. Right: During septic shock, a large number of inflammatory cytokines (including tumour necrosis factor [TNF]-α, interleukin [IL]-1, and IL-6) impair permeability, leading to exudation and enhanced leukocyte recruitment within inflamed tissues.

In septic shock, the immune response and endothelial injury interact. Inflammatory factors released owing to excessive immune activation increase vascular permeability, damage endothelial cells, and cause functional disorders (Figure 3). Endothelial injury further activates the immune response by releasing cytokines and adhesion molecules, recruiting immune cells, and exacerbating local inflammation, thereby creating a vicious cycle. This interaction exacerbates tissue hypoxia and organ failure, thereby affecting patient prognosis.

### 5.1 Endothelial injury induced by immune response

#### 5.1.1 Effects of inflammatory cytokines on endothelial cells

Inflammatory cytokines such as TNF-α, IL-1, and IL-6, which are significantly elevated in septic shock, are the main inducers of endothelial injury. They bind to receptors on endothelial cells and activate downstream signalling pathways that induce endothelial activation and dysfunction, thus promoting endothelial cell damage (Vanden Berghe et al., 2014). For example, TNF-α and IL-1 activate the NF-κB signalling pathway (Liu and Malik, 2006), which promotes endothelial cells to release additional pro-inflammatory cytokines and adhesion molecules such as E-selectin, P-selectin, and intercellular adhesion molecule-1 (ICAM-1), leading to increased adhesion and migration of leukocytes on the vascular endothelium, thereby intensifying the local inflammatory response and forming a vicious cycle (McFadyen et al., 2020). During septic shock, cytokines and NETs degrade and shed the glycocalyx, further recruiting leukocytes to the infection site (Lee et al., 2017).

#### 5.1.2 Endothelial cell apoptosis and damage

Inflammatory cytokines and oxidative stress directly induce endothelial cell apoptosis *via* various mechanisms (Liu and Malik, 2006). Inflammatory cytokines activate intracellular signalling pathways, promoting the expression of inflammatory genes and triggering pro-apoptotic signals (such as caspase-3 activation), which directly cause endothelial cell apoptosis (Tang et al., 2019). These cytokines (e.g., IL-1, IL-6) also activate endothelial cell receptors, induce inflammatory responses, and enhance oxidative stress, which increases vascular permeability, leads to endothelial damage, and promotes apoptosis (Venet and Monneret, 2018). Oxidative stress generates excessive ROS or free radicals that overwhelm the antioxidant system, resulting in damage to cell membranes, proteins, and DNA, thus accelerating endothelial apoptosis. ROS disrupt the stability of cell membranes by oxidising lipids, exacerbating endothelial damage, and promoting apoptosis via signalling pathways (including p38 mitogen-activated protein kinase, Jun N-terminal kinase, and caspases). ROS accumulation also inhibits endothelial repair, further aggravating cell damage (Reid and Webster, 2012).

Inflammatory cytokines and oxidative stress induced by septic shock directly induce endothelial cell apoptosis, compromising the vascular barrier function and increasing vascular permeability. Leakage of plasma components, leukocytes, and cytokines into tissues further intensifies the local and systemic inflammatory responses. This process not only worsens septic shock but also sets the stage for multi-organ failure and death. Therefore, protecting endothelial cells and mitigating the effects of inflammatory cytokines and oxidative stress may serve as crucial strategies for treating septic shock.

### 5.2 Endothelial injury exacerbates immune response

Endothelial cells not only serve as barriers in blood vessels, but also play an essential regulatory role in immune responses. In septic shock, endothelial injury not only results from immune responses but also amplifies the immune response through various mechanisms (Zhang X. et al., 2020).

#### 5.2.1 Increased vascular permeability

Endothelial injury damages tight junctions between endothelial cells, leading to increased vascular permeability (Cui et al., 2014). Immune cells, cytokines, antibodies, and coagulation factors can easily enter the tissues, further extending the local immune response (Cui et al., 2016). The infiltration of immune cells into tissues stimulates the release of inflammatory cytokines, creating a vicious cycle. Endothelial injury not only disrupts vascular barrier function, but also leads to persistent activation of local and systemic immune responses, thereby worsening the condition.

#### 5.2.2 Interaction between leukocytes and endothelial cells

The expression of adhesion molecules such as E-selectin, P-selectin, and ICAM-1 enables immune cells to adhere to the endothelial surface. The widening of inter-endothelial cell gaps allows leukocytes and plasma (containing complements, nutrients, and other components) to pass through the infected vascular wall into the tissues (Reis-Goes et al., 2023). Accumulation of leukocytes on the endothelial surface releases more cytokines and enzymes (such as MMPs), further damaging the structure and function of endothelial cells (Nieuwdorp et al., 2009).

#### 5.2.3 Coagulopathy

Endothelial activation leads to increased release of coagulation factors such as vascular factor VIII, thromboxane, and other procoagulant mediators, as well as changes in NO bioavailability (Arina and Singer, 2021). Endothelial cell damage not only intensifies the immune response, but also activates the coagulation system. In septic shock, endothelial cells release procoagulant factors (e.g., tissue factor), initiating a coagulation cascade and forming microthrombi. The presence of microthrombi further exacerbates local blood flow obstruction, induces organ ischaemia, and promotes the activation of immune cells, creating a more complex immune-coagulation interaction.

### 5.3 Vicious cycle of immune response and endothelial injury

In septic shock, immune response and endothelial injury interact to form a complex bidirectional feedback mechanism. The immune response exacerbates endothelial cell damage through the release of inflammatory cytokines, promoting the progression of septic shock, and endothelial injury increases vascular permeability, activates the coagulation system, and enhances the immune response, creating a vicious cycle (Vincent and De Backer, 2005). This vicious cycle leads to widespread coagulopathy, characterized by dysregulated thrombin generation, fibrin deposition, and microvascular thrombosis. Tissue factor expression on activated endothelial cells and monocytes triggers the extrinsic coagulation pathway, while inflammatory cytokines like TNF-α and IL-6 further amplify this response by downregulating natural anticoagulants such as protein C and antithrombin. Consequently, disseminated intravascular coagulation (DIC) often develops, exacerbating organ hypoperfusion and ischemia through microvascular occlusion. This not only perpetuates endothelial damage but also fuels a pro-inflammatory state, as activated platelets and coagulation factors release additional mediators that recruit more immune cells, thereby intensifying the cycle of injury and coagulopathy. This mechanism not only worsens clinical symptoms, but also affects disease prognosis. Therefore, strategies, such as immune modulation, vascular protection, and anticoagulant therapy, may be effective in alleviating septic shock.

## 6 From basic research to clinical application

Traditional treatments for septic shock primarily rely on antibiotics, haemodynamic support (such as vasoconstrictive agents), and fluid resuscitation (Shi et al., 2020). However, as our understanding of the pathogenesis of septic shock deepens, particularly with regard to immune responses and endothelial injury, treatment strategies have gradually shifted towards immune modulation and endothelial protection. Understanding the interactions between immune responses and endothelial injury is crucial for improving therapeutic outcomes and clinical prognosis. As research in this field progresses, relevant biomarkers have been proposed to support the early diagnosis and prognostic evaluation of septic shock (Skei et al., 2023). For instance, changes in the levels of endothelial markers in the serum (e.g., soluble vascular cell adhesion molecule-1 and soluble E-selectin) may provide valuable information for assessing endothelial injury. Moreover, changes in the plasma levels of inflammatory cytokines can reflect the severity of the immune response and guide clinical treatment strategies.

The translation of basic research into clinical applications hinges on effectively converting the research findings into practical therapeutic strategies. Several translational studies focused on the interaction between immune responses and endothelial injury in septic shock have already shown progress (Han et al., 2015).

### 6.1 Immune modulation therapy

Immune modulation therapy has become another important therapeutic approach owing to the overly intense immune response in septic shock (Hotchkiss and Opal, 2020). Immunomodulatory therapy utilising anti-inflammatory agents or immune checkpoint inhibitors may not only enhance the body’s immune function but also improve the prognosis of sepsis and septic shock. Studies have shown that anti-inflammatory factors (e.g., IL-10, TGF-β) can effectively attenuate the pathological process of septic shock (Han et al., 2015), and immune checkpoint inhibitors such as TIM-3, PD-1, and B- and T-lymphocyte attenuator can restore the function of innate and acquired immune cells and reverse the state of immune depletion. Tocilizumab, an IL-6 receptor antagonist, has demonstrated clinical benefits in a subset of patients with septic shock, particularly in controlling systemic inflammatory responses (Reisinger et al., 2020). By blocking the IL-6 receptor, tocilizumab inhibits downstream pro-inflammatory cascades, thereby modulating cytokine release syndrome (CRS). Research indicates that tocilizumab effectively reduces the levels of inflammatory markers, including IL-6, IL-8, and monocyte chemoattractant protein-1, and attenuates vascular endothelial cell activation in patients with CRS (Kang et al., 2020). Notably, tocilizumab may increase the risk of infection in immunocompromised patients. However, studies have shown that tocilizumab does not significantly increase the incidence of bloodstream infections when managing CRS severity (De Togni et al., 2022). This suggests that meticulous assessment of patients’ infection risk and inflammatory status is essential to optimise therapeutic outcomes when administering tocilizumab. Immunoglobulin therapy may improve outcomes in patients with sepsis and multidrug-resistant (MDR) bacterial infections. IgM-enriched immunoglobulin (IgGAM) demonstrates significant efficacy against severe infections caused by MDR Gram-negative bacteria. In a study of 100 patients, the IgGAM-treated group exhibited significantly lower 28-day all-cause mortality than controls (p = 0.011), with notably reduced mortality in sepsis caused by extensively drug-resistant (XDR) pathogens (p = 0.008) (Giamarellos-Bourboulis et al., 2016). This suggests that IgM-enriched immunoglobulin is an effective adjunctive therapy to antibiotics for improving outcomes in sepsis induced by MDR Gram-negative bacteria. However, not all studies have supported the efficacy of immunoglobulins in sepsis management. A retrospective investigation examining polyspecific intravenous immunoglobulin (IVIG) in patients having sepsis with XDR pathogens found no significant differences in the 30-day mortality (p = 0.886) or length of intensive care unit (ICU) stay between the IVIG-treated and control groups (Acar et al., 2025). This implies that while immunoglobulins may provide benefits in specific scenarios, their therapeutic value in XDR pathogen-induced sepsis requires further validation. Collectively, these findings suggest that immunoglobulin therapy may offer potential benefits for select patients with sepsis, although its effectiveness appears to be contingent on individual clinical profiles and pathogen characteristics (Beudeker et al., 2022). Thymosin α1 activates innate immune cells such as dendritic and natural killer cells, whereas macrophages stimulate T-cell proliferation and enhance the antimicrobial effects of Th1 cells (Li et al., 2015). Mesenchymal stem cells (MSCs) promote the maturation of M2-type macrophages and regulate T-cell maturation, thereby facilitating bacterial clearance, limiting excessive inflammation, attenuating organ damage, and ultimately reducing septic shock mortality (Pan et al., 2022). In addition, corticosteroids and other immunosuppressive agents have demonstrated significant efficacy in modulating the inflammatory responses (Zou et al., 2023). IV corticosteroid administration constitutes a key recommendation in the Surviving Sepsis Campaign (SSC) guidelines. According to the 2021 SSC update, corticosteroid therapy is recommended when haemodynamic stability cannot be maintained despite adequate fluid resuscitation and vasopressor support (Prescott and Ostermann, 2023). This recommendation acknowledges the potential benefits of corticosteroids in improving outcomes for patients with septic shock, although their associated adverse effects require vigilant monitoring. Studies indicate that systemic administration of high-dose corticosteroids effectively counters acute rejection episodes but concurrently incurs profound side effects (van Alem et al., 2020). For instance, in pulmonary sarcoidosis management, although corticosteroids resolve granulomatous inflammation, patients may develop severe complications such as fibrosis and other treatment-related morbidities (Judson, 2019). Novel therapeutic strategies, particularly targeted drug delivery systems, may maintain clinical benefits while reducing adverse events, thereby enhancing patients’ quality of life. Recent advances in the understanding of the pathophysiology of septic shock have positioned haemoadsorption therapy as a promising immunomodulatory approach. By removing key inflammatory mediators from circulation, this intervention may improve patient outcomes. First, haemoadsorption demonstrates potential benefits in optimising fluid balance and preserving endothelial function. A study revealed significantly reduced fluid balance within 72 h in patients receiving CytoSorb® therapy compared to non-survivors, suggesting enhanced endothelial integrity (Kogelmann et al., 2024). Furthermore, CytoSorb® treatment reduced catecholamine requirements and lowered in-hospital mortality, supporting its therapeutic potential in septic shock management (Rugg et al., 2020). Second, early intervention is critical for efficacy. A single-centre analysis of 175 patients with septic shock found that early CytoSorb® initiation (≤24 h post-diagnosis) correlated with more pronounced haemodynamic stabilisation and reduced ICU mortality (Berlot et al., 2025). Notably, treatment intensity, rather than timing alone, was associated with improved survival, suggesting aggressive early haemoadsorption in refractory cases. Despite these encouraging findings, their clinical application requires further investigation. Although current evidence suggests context-dependent benefits, rigorously designed trials are needed to establish efficacy across diverse patient populations.

### 6.2 Endothelial protection therapy

Endothelial cell injury is a crucial pathological feature of septic shock, and protecting endothelial function has become a focus of research in the treatment of septic shock. For example, antioxidants (e.g., vitamin C and lipoic acid) can mitigate oxidative stress, block glycocalyx shedding, and protect endothelial cells from damage, with positive results in some clinical studies. Vitamin C, which serves as both an antioxidant and a critical cofactor for neutrophil function, demonstrates anti-inflammatory properties in sepsis. Although mechanistic evidence suggests potential benefits *via* antioxidant effects and endothelial glycocalyx protection (e.g., reducing syndecan-1), pivotal clinical trials (LOVIT, VICTAS) revealed that high-dose intravenous vitamin C (≥50 mg/kg/day) increased 28-day mortality and organ failure risk (LOVIT: NNH = 9) while failing to improve hard endpoints (survival/organ function). Nevertheless, the endothelioprotective potential of antioxidants remains significant and warrants further investigation (W et al., 2023). Recombinant human thrombopoietin can reduce the levels of inflammatory factors (such as IL-6 and TNF-α) associated with endothelial injury and mitigate endothelial cell damage in diseases like septic shock (Zhang J. et al., 2020). Specific therapies targeting leukocyte adhesion, such as antibodies against adhesion molecules like CD11a, have proven effective in sepsis-induced lung injury (Wang et al., 2013). Recombinant human lipoprotein-associated phospholipase A2 receptor has shown the potential to restore endothelial function in some clinical trials (Ahn et al., 2017). Moreover, researchers are exploring drugs that modulate the connections between endothelial cells (such as vascular endothelial growth factor receptor antagonists and Ras homolog gene family member A/Rho-associated protein kinase pathway inhibitors) to restore vascular barrier function (Zhao et al., 2023).

### 6.3 Immune endothelial axis repair and precision therapy

#### 6.3.1 Immunomodulation: from broad-spectrum suppression to precision stratification

Regarding immunotype-guided corticosteroid therapy, a secondary analysis of the ADRENAL trial (n = 540) classified patients with septic shock into immuno-adaptive phenotype (IA-P) and immuno-naive phenotype (IN-P). The study found that patients with IA-P (especially those with pulmonary infections) had a significantly increased 28-day mortality risk with hydrocortisone (OR, 5.55), while those with IN-P potentially benefited (Venkatesh et al., 2025). Advances also emerge in novel immunomodulators; multiple Phase II trials focus on TNF-α/IL-1β monoclonal antibodies (e.g., CDP-571) and Janus kinase inhibitors (e.g., tofacitinib), aiming to suppress early cytokine storms while balancing immunosuppression against pathogen clearance (Sepsis: A Molecular Odyssey from Infection to Organ Failure). Breakthroughs have occurred in agents targeting CLEC5A (expressed on endothelial cells), a key receptor driving leukocyte infiltration. Its monoclonal antibody (mAb-13) reduces pulmonary vascular leakage by 50% in animal models and has entered Phase Ib trials (NCT05211439) (Zhang et al., 2025). In cell therapy and metabolic interventions, MSCs repair tissue damage by modulating macrophage polarisation (M1→M2) and mitochondrial transfer. The Phase III REALIST trial evaluated MSCs for the treatment of sepsis-induced ARDS (Sepsis: A Molecular Odyssey from Infection to Organ Failure). The vitamin B5 derivative dexpanthenol reduced TNF-α/IL-1β by 40% in LPS-induced shock models and mitigated oxidative stress *via* glutathione elevation, now in Phase II dose-finding trials (NCT04156988) (Protective effect of dexpanthenol in a rat model of LPS-induced endotoxic shock).

#### 6.3.2 Endothelial barrier protection: from passive support to active repair

Regarding connexin stability regulation, annexin A3 deficiency reduces VE-cadherin/ZO-1 expression, exacerbating vascular leakage. Its small-molecule agonist stabilises the actin cytoskeleton, reducing pulmonary oedema by 60% in animal models, and Phase I trials are planned for 2026 (Xing et al., 2025). ARF6 inhibitors enhance endothelial junctions by restoring VE-cadherin membrane localisation while inhibiting inflammasome propagation Phase III trials showed a 22% relative reduction in 28-day mortality for sepsis-induced lung injury (Oshima et al., 2025). For glycocalyx repair, sulodexide (a glycosaminoglycan complex) inhibits glycocalyx-degrading enzymes and upregulates tight junction proteins *via* the NF-κB/ZO-1 pathway. The CLOVERS trial subgroup analysis revealed that its combination with restrictive fluid resuscitation increased the endothelial dysfunction reversal rates by 35% (Oshima et al., 2025). Syndecan-1 ectodomain replacement therapy reduced vascular leakage markers (e.g., Ang-2) by >50% in Phase I trials (NCT04879251) (Oshima et al., 2025). Table 1 summarises the aforementioned results.

**TABLE 1:** Key clinical trials summary in septic shock management.

Trial name/Agent	Target/Mechanism	Development stage	Key findings/Conclusions
Immunomodulation			
Immunotype-guided Steroid Therapy (ADRENAL sub-analysis)	Genotype-based stratification (IA-P/IN-P)	Retrospective analysis (n = 540)	↑28-day mortality risk in IA-P (OR 5.55), especially pulmonary infections; Potential benefit in IN-P
CLEC5A mAb (mAb-13)	Blocks endothelial CLEC5A receptor	Phase Ib (NCT05211439)	↓50% pulmonary vascular leakage in animal models; Reduces neutrophil infiltration
Mesenchymal Stem Cells (REALIST Trial)	Modulates macrophage polarization (M1→M2)	Phase III (Ongoing)	Repairs tissue damage *via* mitochondrial transfer; Evaluates efficacy in ARDS
Vitamin B5 Derivative (Dexpanthenol)	↑GSH to mitigate oxidative stress	Phase II (NCT04156988)	↓40% TNF-α/IL-1β in LPS models
Endothelial protection			
ANXA3 Agonist (ANX-102)	Stabilizes actin cytoskeleton; ↑VE-cadherin/ZO-1	Preclinical → Phase I Prep (2026)	↓60% pulmonary edema in animal models
ARF6 Inhibitor (NAV-2729)	Restores VE-cadherin membrane localization	Phase III Completed	↓22% 28-day mortality in septic lung injury; Suppresses inflammasome propagation
Sulodexide	Inhibits glycocalyx degradation; ↑ZO-1 *via* NF-κB pathway	Phase II (CLOVERS subgroup)	↑35% endothelial dysfunction reversal rate when combined with restrictive fluid resuscitation
Syndecan-1 Replacement Therapy (rSDC1)	Recombinant syndecan-1 ectodomain	Phase I (NCT04879251)	↓>50% vascular leakage markers (e.g., Ang-2)

In summary, septic shock treatment is undergoing a revolution regarding decentralisation, pivoting from monotherapy anti-infection approaches towards dual-axis immune-endothelial modulation. Future management requires precise phenotyping, multi-target drug combinations, and real-time monitoring to achieve the closed-loop restoration of endothelial integrity, immune equilibrium, and systemic homeostasis.

### 6.4 Microcirculation restoration therapy

Microcirculatory dysfunction is a hallmark of septic shock. Even after receiving optimal haemodynamic support (including fluid resuscitation, vasopressors, and cardiac agents), some patients exhibit microcirculatory impairments. Therefore, improving microcirculation has become a crucial therapeutic goal (Satoshi Gando et al., 2013; Hellman et al., 2021; Legrand et al., 2020). Recently, multiple drugs have demonstrated the potential to enhance microcirculation and restore blood flow. Ilomedin, a prostacyclin analogue with vasodilatory and antithrombotic properties that is particularly effective at the microcirculatory level, has shown promise. Studies indicate that Early intravenous administration of ilomedin in patients with persistent microcirculatory perfusion impairment during septic shock can improve organ function recovery (Legrand et al., 2020). In contrast, although iloprost does not reduce the severity of organ failure in patients with chronic hypoperfusion, preliminary preclinical and clinical data suggest that it may enhance tissue perfusion. This suggests that the ongoing research on drug-mediated microcirculation improvement is supported by theoretical evidence (Legrand et al., 2025). In addition to these drugs, other substances have also been explored. Research has revealed that resveratrol improves liver function and hepatocyte integrity in rats with haemorrhagic shock, with some effects mediated by oestrogen receptors. Although this study was mainly aimed at haemorrhagic shock, it also suggested the possibility of some substances improving organ perfusion and function, providing a new idea for finding drugs to improve microcirculation blood flow (Wolf et al., 2022).

### 6.5 Personalized treatment strategies

With based on a deeper understanding of the molecular mechanisms of septic shock are gradually becoming possible (Hotchkiss et al., 2013). By precisely measuring indicators such as the immune response, extent of endothelial injury, and microcirculatory function, clinicians can adjust treatment plans according to a patient’s specific pathological state. Although direct studies on immune responses are limited, research has highlighted the importance of biomarkers for sepsis and septic shock treatment. These biomarkers provide insights into the complex pathophysiological processes, enabling more precise therapeutic approaches (von Groote and Meersch-Dini, 2022). Immune response-related biomarkers may be the key components. By measuring these indicators (e.g., C-reactive protein, Procalcitonin, Presepsin [soluble CD14], IL-6, IL-8, neutrophil CD64 expression, soluble programmed death ligand 1, human leukocyte antigen DR isotype [HLA-DR] expression on antigen-presenting cells, pentraxin, and complement protein 5a), clinicians can better assess patients’ immune status and adjust treatment regimens accordingly. Measuring the extent of endothelial damage has significant clinical value. Studies evaluating endothelial biomarkers in ICU admissions of patients with septic shock have revealed that angiogenesis factor-2 correlates with clinical severity, whereas endocan can predict the occurrence of respiratory failure by day 3 (Palud et al., 2015). These findings demonstrate that measuring endothelial biomarkers provides clinicians with critical information regarding the risk of organ failure, enabling more targeted treatment strategies. Regarding microcirculation assessment, research indicates that evaluating peripheral perfusion index and perfusion vascular velocity ratio can predict organ dysfunction and 28-day mortality in septic shock patients (Pan et al., 2018). Notably, studies have identified the proportion of perfused small vessels as the strongest prognostic factor, showing a significant correlation between disease severity and survival rate (De Backer et al., 2013). Furthermore, microvascular perfusion density is strongly associated with organ dysfunction and mortality, whereas hyperlactatemia and increased norepinephrine demand increase the likelihood of severe microvascular impairments (Hernandez et al.). These findings strongly support the importance of the precise measurement of microcirculatory parameters to assess patient severity and guide therapeutic adjustments.

## 7 Future research directions

In the transition from basic research to clinical applications for septic shock, future research will increasingly emphasise the application of precision medicine and advance personalised treatment to a deeper level. In recent years, with the continuous progress in multi-omics technologies, such as genomics, proteomics, and metabolomics, researchers are now able to more precisely reveal the molecular mechanisms of septic shock and explore individual differences (Schuurman et al., 2021). These differences are not only reflected at the genomic level in terms of genetic susceptibility, but also manifest in immune system responses, endothelial function, and microcirculatory performance. Therefore, future research should focus on the use of these high-throughput technologies to obtain individualised biomarkers to tailor the most appropriate treatment plans for each patient (Cavaillon et al., 2020).

Overall, the treatment of septic shock will evolve towards being more precise, efficient, and personalised. By further studying the molecular mechanisms, developing novel targeted therapies, and optimising immune modulation and endothelial protection strategies, we hope to achieve better clinical outcomes, reduce the mortality rate of septic shock, and improve patients’ quality of life.

## 8 Discussion

Sepsis is a life-threatening clinical syndrome defined as acute organ dysfunction triggered by a dysregulated host response to infection. This maladaptive response involves complex interactions between pathogen-derived factors and immune cell-mediated inflammatory cascades, which may lead to adverse outcomes throughout the disease progression. Septic shock, the most severe manifestation, is characterised by profound circulatory, cellular, and metabolic derangements, conferring a substantially higher mortality risk than sepsis alone (Ferrario et al., 2019; Shankar-Hari et al., 2016).

When the body encounters a severe infection, the immune system initiates an inflammatory response by releasing cytokines and chemokines to eliminate pathogens. However, an excessive immune response, resulting in a cytokine storm, not only promotes the recruitment and activation of immune cells, but also directly damages endothelial cells, impairing their barrier function. This leads to oedema, hypotension, and microcirculatory dysfunction (Chang, 2019). These haemodynamic changes exacerbate tissue hypoxia and metabolic disturbances, ultimately resulting in organ failure. Overactivation of the immune response may cause immune suppression, particularly in the later stages of septic shock. Suppression of the immune system makes the body more vulnerable to secondary infections, creating a vicious cycle that further aggravates the condition.

The vicious cycle of immune-endothelial interactions is not only a crucial mechanism for the onset and progression of septic shock but is also a significant challenge in clinical treatment. The current treatment strategies mainly focus on antimicrobial therapy, haemodynamic support, and immune modulation. As our understanding of the mechanisms underlying endothelial injury and immune response interactions increases, new therapeutic targets continue to emerge. Researchers are exploring treatment strategies that target endothelial cell function, improve vascular permeability, and repair microcirculation, including vascular protectants, anti-inflammatory cytokines, and endothelial function-restoring agents. Similarly, in clinical practice, the early identification of biomarkers indicating endothelial injury and immune dysfunction in patients with septic shock can provide critical guidance for treatment planning. Targeted intervention with these key factors may effectively slow down immune system overactivation or suppression, protect endothelial cells, restore vascular function, and ultimately improve patient survival and quality of life.

However, owing to the complexity of the immune response and vascular damage mechanisms, existing treatments often fail to effectively address the pathophysiological issues in patients with septic shock. Consequently, an increasing number of researchers are focusing on precisely intervening in immune responses and endothelial injury to break this vicious cycle, with the aim of improving treatment outcomes.

In summary, the interaction between endothelial injury and the immune response is not only a core mechanism of septic shock, but also a key breakthrough point for future therapies. With the advancement of basic research and clinical innovations, we hope to overcome existing treatment bottlenecks and provide more precise and efficient therapeutic strategies, ultimately improving the survival rates and quality of life of patients with septic shock.
